# Validity of Self-Reported Height and Weight in a Korean Population

**DOI:** 10.2188/jea.JE20100058

**Published:** 2011-01-05

**Authors:** Dong-Hun Lee, Aesun Shin, Jeongseon Kim, Keun-Young Yoo, Joohon Sung

**Affiliations:** 1 Cancer Epidemiology Branch, National Cancer Center, Goyang-si, Korea; 2 Department of Epidemiology, School of Public Health and Institute of Health and Environment, Seoul National University, Seoul, Korea; 3 Department of Preventive Medicine, Seoul National University College of Medicine, Seoul, Korea

**Keywords:** validity, height, weight, body mass index, self-report

## Abstract

**Background:**

Accessible public information on self-reported height and weight is not widely used in studies of obesity, mainly because of the questionable validity of body mass index (BMI) values calculated from these data. To assess the utility of self-reported measurement, we compared self-reported and standard measurements of height and weight in a Korean population that is leaner than Western populations.

**Methods:**

A cross-sectional comparison of self-reported and measured height and weight was conducted among a population of participants in a cancer screening program. A total of 557 men and 1010 women aged 30 to 70 years were included in the current analysis.

**Results:**

Self-reported height was higher than measured values in both men and women. Self-reported weight was higher than measured weight in women, but was not different in men. BMI calculated from measured values was higher than BMI derived from self-reported height and weight among men. Younger age was a predictor of accuracy in self-reported height, and higher weight and BMI were predictors of under-reporting of weight. The prevalence of obesity based on self-reported values was lower than the true prevalence of obesity. With respect to classifying individuals as obese, the specificity and sensitivity of BMI calculated from self-reported values were very high for both sexes.

**Conclusions:**

Self-reported height and weight were reasonably valid in this study population.

## INTRODUCTION

Height and weight are the most commonly used anthropometric measurements in clinical practice and research, and are of interest in epidemiological studies, both as primary exposures and as potential confounding variables. Height and weight are common, straightforward components of nutritional status assessments because they are strong predictors of functional impairment, morbidity, and mortality. They are also valuable for assessments of the overall health status of older adults.^1^^–^^5^

Body mass index (BMI) is calculated from height and weight and can be used to assess nutritional status and overall health outcomes such as obesity. Obesity has been identified as an important risk factor for many diseases, including cardiovascular disease, diabetes, and some cancers.^6^^–^^10^

Self-reported height and weight are commonly used in epidemiological studies because they provide a simple, inexpensive, and quick method of collecting data from large populations. In previous studies, self-reported height and weight were shown to have acceptable accuracy.^11^^–^^13^ In addition, most studies have shown a high correlation between self-reported and measured height and weight.^12^^,^^14^^–^^16^

Because of limited resources, self-reported data may be the only information available. Several demographic and health characteristics have been shown to be predictors of the accuracy of self-reported height and weight. Accessible public data on self-reported height and weight are not widely used in studies of obesity, mainly because the validity of BMI calculated from these data is questionable. For example, men are more likely to overestimate their height,^14^ and women are more likely to underestimate their weight.^15^^,^^16^ Overweight people have a greater tendency to under-report their weight than do people of lower weight, especially women.^14^^–^^18^ In a study conducted in a Korean population, however, weight was overestimated in both men and women.^19^

Many adults, especially older adults, may be unaware of their actual height and weight.^20^^,^^21^ Older men tended to overestimate their height more often than did younger men.^12^^,^^14^^,^^15^^,^^20^^,^^22^ Overestimation of height can result in the calculation of a lower BMI value, due to the higher denominator. One study showed that the sensitivity of a categorical definition of obesity (defined as BMI ≥30) based on self-reported height and weight substantially decreased with increasing age among both men and women.^23^ Thus, BMI calculated from self-reported height and weight may result in misclassification of individuals with respect to their risk of medical problems and their need for dietary, lifestyle, and medical interventions.

The objective of this study was to assess the validity of self-reported height and weight by comparing those values with measured values as the gold standard in a Korean population that is leaner than Western populations. In addition, we evaluated whether BMI calculated from self-reported height and weight was suitable for use in public health practice and research.

## METHODS

### Subjects

The study participants were adults aged 30 to 70 years who underwent a health examination at the Center for Early Detection and Prevention, National Cancer Center, Korea between 1 October 2007 and 31 December 2008. During this period, 1731 of 3303 examinees agreed to participate in the study (response rate: 52.4%). After individuals with incomplete data for self-reported height and weight were excluded (*n* = 164), 557 men and 1010 women remained for analysis. The study protocol was approved by the Institutional Review Board of the National Cancer Center.

### Questionnaire

A week before the scheduled health examination, examinees were sent and asked to complete a questionnaire that collected information on demographics, lifestyle factors including cigarette smoking, dietary habits, and medical history, and current height and weight. On the day of the health examination, interviewers met the examinees at the hospital, obtained informed consent, and collected the completed questionnaire. The responses to questions about current height and weight were used as the self-reported values in the analysis.

### Measurements

Height and weight were measured during the health examination by trained hospital nurses. Height was measured to the nearest 0.1 cm with an optical linear encoder scale (DS-102; Dongsahn Jenix Co, Seoul, Korea); weight was measured to the nearest 0.1 kg with an electronic load cell scale (DS-102; Dongsahn Jenix Co, Seoul, Korea). Height and weight were measured while participants wore light indoor clothes and no shoes. BMI was calculated as weight in kilograms divided by height in meters squared.

### Statistical analysis

Student’s paired samples *t* test was used to compare the means of self-reported and measured values. Pearson’s correlation coefficient was used for evaluating concordance between self-reported and measured values. We calculated mean differences between self-reported and measured height, weight, and BMI by quartile of measured height, quartile of measured weight, 4 categorical groups for BMI (<20, 20–24.9, 25–29.9, ≥30 kg/m^2^), age group (<45, 45–49, 50–54, ≥55 years), education level (middle school, high school, junior college, college graduate), smoking status (never smoker; ex-smoker who had quit at least 1 year before; current smoker, including ex-smokers who quit smoking within the previous 1 year), and disease history (hypertension, diabetes mellitus, hyperlipidemia). We also cross-tabulated self-reported values with measured values in quartiles for height, weight, BMI, and the 4 categorical groups for BMI (<20, 20–24.9, 25–29.9, ≥30 kg/m^2^). In the 4 × 4 contingency tables for self-reported and measured values, Kappa statistics were calculated to assess the degree of concordance. *P* for trend was calculated using linear regression. The order of categorical variables was an independent variable and the differences between self-reported and measured values were dependent variables. Participants with a BMI ≥25.0 kg/m^2^ were defined as obese, based on the obesity criterion proposed for Asian populations.^24^ Prevalence of obesity based on self-reported BMI was compared with measured BMI. Sensitivity, specificity, positive predictive value, and negative predictive value were used to assess the validity of determining obesity based on self-reported data. All statistical analyses were performed using SAS (version 9.1.3; SAS Institute Inc, Cary, NC, USA).

## RESULTS

The mean (standard deviation) age of the study population was 52.3 (9.41) years in men and 50.2 (8.23) years in women.

In both men and women, there were strong correlations (>0.9) between self-reported and measured values for height, weight, and BMI (Table 1). Self-reported height was higher than measured height by 0.41 cm (95% confidence interval [CI]: 0.27–0.55) in men and 0.51 cm (0.39–0.63) in women. Likewise, the mean differences for weight were 0.12 kg (−0.04 to 0.28) in men and 0.26 kg (0.16–0.36) in women. In contrast, BMI calculated using measured height and weight was higher than that calculated using self-reported values: the mean difference was −0.08 kg/m^2^ (−0.14 to −0.02) in men and −0.05 kg/m^2^ (−0.11 to 0.01) for women.

**Table 1.** Means (standard deviations), correlations, and differences of self-reported and measured anthropometric indices

Variable	Self-reported	Measured	Pearson’s correlation coefficient	Difference (95% CI)
Men (*n* = 557)				
Height (cm)	169.98 (5.53)	169.57 (5.63)	0.956^b^	0.41^b^ (0.27, 0.55)
Weight (kg)	70.96 (8.96)	70.84 (9.03)	0.978^a^	0.12 (−0.04, 0.28)
BMI (kg/m^2^)	24.53 (2.62)	24.61 (2.70)	0.953^b^	−0.08^a^ (−0.14, −0.02)
Women (*n* = 1010)				
Height (cm)	157.95 (4.95)	157.43 (5.05)	0.918^b^	0.51^b^ (0.39, 0.63)
Weight (kg)	57.68 (7.09)	57.42 (7.28)	0.980^b^	0.26^b^ (0.16, 0.36)
BMI (kg/m^2^)	23.13 (2.72)	23.17 (2.82)	0.953^b^	−0.05 (−0.11, 0.01)

Difference = (self-reported value − measured value).

^a^*P* < 0.05; ^b^*P* < 0.001.

Abbreviations: CI, confidence interval; BMI, body mass index.

In both men and women, older age was associated with overestimation of self-reported height (Table 2). As a result, BMI calculated based on self-reported data was lower in older men (*P* for trend = 0.024) and in older women (*P* for trend = 0.003). In both men and women, higher BMI was associated with overestimation of self-reported height and underestimation of self-reported weight and, consequently, with underestimated BMI. Interestingly, lower quartiles of measured height were associated with overestimation of self-reported height and, consequently, underestimated BMI. In both men and women, overestimation of self-reported height was associated with lower educational attainment. Smoking status did not affect the difference between self-reported and measured anthropometrics values in men or women. The presence of hypertension was associated with a greater difference between self-reported and measured height in both men and women: men with hypertension were more likely to overestimate their height. Men with diabetes were more likely to underestimate their weight and BMI, but women with diabetes were not. The presence of hyperlipidemia was not associated with differences between self-reported and measured values in men or women. Adjusting for age (for BMI) or age and BMI (for weight and height) did not substantially change the results (data not shown).

**Table 2.** Mean differences between self-reported and measured anthropometric indices by age group, quartile of measured height, quartile of measured weight, and BMI group

Category	*n*	Height		Weight		BMI	
		
		Difference (95% CI)	*P*	Difference (95% CI)	*P*	Difference (95% CI)	*P*
Men (*n* = 557)							
Age group, years							
<45	128	0.21^a^ (0.01, 0.41)	0.039^c^	0.11 (−0.16, 0.38)	0.992^c^	−0.02 (−0.14, 0.10)	0.235^c^
45–49	77	0.25 (−0.18, 0.68)		0.13 (−0.24, 0.50)		−0.04 (−0.24, 0.16)	
50–54	133	0.47^a^ (0.20, 0.74)		0.11 (−0.28, 0.50)		−0.10 (−0.26, 0.06)	
≥55	219	0.55^b^ (0.31, 0.79)		0.12 (−0.12, 0.36)		−0.12^a^ (−0.24, 0.00)	
Highest educational attainment							
Less than high school	80	0.95^b^ (0.46, 1.44)	0.002^c^	0.28 (−0.19, 0.75)	0.133^c^	−0.19 (−0.43, 0.05)	0.508^c^
High school	140	0.51^b^ (0.24, 0.78)		0.30 (−0.07, 0.67)		−0.05 (−0.19, 0.09)	
Some college	60	0.15 (−0.14, 0.44)		−0.10 (−0.59, 0.39)		−0.07 (−0.25, 0.11)	
College graduate	277	0.26^a^ (0.08, 0.44)		0.03 (−0.15, 0.21)		−0.07 (−0.15, 0.01)	
Smoking status							
Never smoker	116	0.52^a^ (0.17, 0.87)	0.627^d^	0.28^a^ (0.01, 0.55)	0.572^d^	−0.07 (−0.23, 0.09)	0.842^d^
Ex-smoker (quit longer than 1 year)	253	0.42^b^ (0.24, 0.60)		0.06 (−0.19, 0.31)		−0.11^a^ (−0.21, −0.01)	
Current smoker	188	0.33^a^ (0.08, 0.58)		0.09 (−0.16, 0.34)		−0.06 (−0.18, 0.06)	
Present hypertension							
No	438	0.31^b^ (0.17, 0.45)	0.006^e^	0.12 (−0.06, 0.30)	0.934^e^	−0.05 (−0.13, 0.03)	0.062^e^
Yes	119	0.78^b^ (0.41, 1.15)		0.11 (−0.18, 0.40)		−0.21^a^ (−0.37, −0.05)	
Present diabetes							
No	516	0.39^b^ (0.25, 0.53)	0.300^e^	0.18^a^ (0.02, 0.34)	0.003^e^	−0.05 (−0.13, 0.03)	0.003^e^
Yes	41	0.67^a^ (0.26, 1.08)		−0.72^a^ (−1.37, −0.07)		−0.45^b^ (−0.70, −0.20)	
Present hyperlipidemia							
No	476	0.41^b^ (0.25, 0.57)	0.989^e^	0.11 (−0.07, 0.29)	0.742^e^	−0.08^a^ (−0.16, 0.00)	0.888^e^
Yes	81	0.41^a^ (0.10, 0.72)		0.18 (−0.19, 0.55)		−0.07 (−0.25, 0.11)	
Quartile of measured height, cm							
<165	134	0.66^b^ (0.33, 0.99)	<0.001^c^	0.25 (0.00, 0.50)	0.988^c^	−0.10 (−0.24, 0.04)	0.030^c^
165–168.9	104	0.86^b^ (0.47, 1.25)		−0.10 (−0.45, 0.25)		−0.29^a^ (−0.47, −0.11)	
169–172.9	159	0.28^a^ (0.06, 0.50)		0.06 (−0.16, 0.28)		−0.06 (−0.16, 0.04)	
≥173	160	0.04 (−0.16, 0.24)		0.20 (−0.17, 0.57)		0.05 (−0.09, 0.19)	
Quartile of measured weight, kg							
<65	125	0.22 (−0.07, 0.51)	0.592^c^	0.47^a^ (0.20, 0.74)	0.012^c^	0.11 (−0.03, 0.25)	<0.001^c^
65–69.9	134	0.60^b^ (0.36, 0.84)		0.00 (−0.27, 0.27)		−0.18^a^ (−0.30, −0.06)	
70–75.9	153	0.40^b^ (0.20, 0.60)		0.25 (−0.10, 0.60)		−0.04 (−0.18, 0.10)	
≥76	145	0.41^a^ (0.06, 0.76)		−0.22 (−0.51, 0.07)		−0.21^a^ (−0.37, −0.05)	
BMI category, kg/m^2^							
<20	21	−0.14 (−0.57, 0.29)	<0.001^c^	1.38^a^ (0.54, 2.22)	<0.001^c^	0.53^a^ (0.20, 0.86)	<0.001^c^
20–24.9	302	0.24^a^ (0.08, 0.40)		0.22^a^ (0.00, 0.44)		0.01 (−0.07, 0.09)	
25–29.9	217	0.59^b^ (0.37, 0.81)		−0.10 (−0.34, 0.14)		−0.21^b^ (−0.31, −0.11)	
≥30	17	1.88 (−0.22, 3.98)		−0.47 (−1.10, 0.16)		−0.8^a^ (−1.51, −0.09)	

Women (*n* = 1010)							
Age groups, years							
<45	285	0.13 (−0.09, 0.35)	<0.001^c^	0.16 (−0.02, 0.34)	0.102^c^	0.03 (−0.07, 0.13)	0.002^c^
45–49	224	0.30^b^ (0.14, 0.46)		0.21^a^ (−0.01, 0.43)		0.00 (−0.10, 0.10)	
50–54	216	0.48^b^ (0.26, 0.70)		0.30^b^ (0.12, 0.48)		−0.02 (−0.12, 0.08)	
≥55	285	1.08^b^ (0.77, 1.39)		0.35^b^ (0.19, 0.51)		−0.19^a^ (−0.31, −0.07)	
Highest educational attainment							
Less than high school	179	1.03^b^ (0.60, 1.46)	<0.001^c^	0.25^a^ (0.05, 0.45)	0.099^c^	−0.21^a^ (−0.37, −0.05)	0.052^c^
High school	406	0.55^b^ (0.37, 0.73)		0.38^b^ (0.24, 0.52)		−0.01 (−0.09, 0.07)	
Some college	95	0.42^a^ (0.17, 0.67)		0.14 (−0.10, 0.38)		−0.07 (−0.19, 0.05)	
College graduate	330	0.21^a^ (0.01, 0.41)		0.14 (−0.04, 0.32)		0.00 (−0.10, 0.10)	
Smoking status							
Never smoker	938	0.55^b^ (0.41, 0.69)	0.014^d^	0.26^b^ (0.16, 0.36)	0.163^d^	−0.06^a^ (−0.12, 0.00)	0.018^d^
Ex-smoker (quit longer than 1 year)	33	−0.48 (−1.52, 0.56)		0.50^a^ (0.13, 0.87)		0.34^a^ (0.01, 0.67)	
Current smoker	39	0.38 (−0.05, 0.81)		−0.13 (−0.48, 0.22)		−0.18^a^ (−0.36, 0.00)	
Present hypertension							
No	849	0.45^b^ (0.31, 0.59)	0.017^e^	0.23^b^ (0.13, 0.33)	0.186^e^	−0.04 (−0.10, 0.02)	0.305^e^
Yes	161	0.86^b^ (0.57, 1.15)		0.39^b^ (0.19, 0.59)		−0.11 (−0.25, 0.03)	
Present diabetes							
No	956	0.49^b^ (0.35, 0.63)	0.197^e^	0.26^b^ (0.16, 0.36)	0.815^e^	−0.04 (−0.10, 0.02)	0.213^e^
Yes	54	0.86^b^ (0.47, 1.25)		0.21 (−0.12, 0.54)		−0.19 (−0.41, 0.03)	
Present hyperlipidemia							
No	886	0.48^b^ (0.34, 0.62)	0.155^e^	0.25^b^ (0.15, 0.35)	0.616^e^	−0.04 (−0.10, 0.02)	0.467^e^
Yes	124	0.75^b^ (0.44, 1.06)		0.32^a^ (0.05, 0.59)		−0.10 (−0.24, 0.04)	
Quartile of measured height, cm							
<154	214	1.18^b^ (0.87, 1.49)	<0.001^c^	0.22^a^ (0.04, 0.40)	0.763^c^	−0.27^b^ (−0.41, −0.13)	<0.001^c^
154–157.9	288	0.74^b^ (0.45, 1.03)		0.29^b^ (0.13, 0.45)		−0.09 (−0.19, 0.01)	
158–160.9	233	0.31^b^ (0.19, 0.43)		0.32^a^ (0.10, 0.54)		0.03 (−0.07, 0.13)	
≥161	275	−0.07 (−0.29, 0.15)		0.19^a^ (0.03, 0.35)		0.09^a^ (−0.01, 0.19)	
Quartile of measured weight, kg							
<53	251	0.41^b^ (0.19, 0.63)	0.732^c^	0.61^b^ (0.45, 0.77)	<0.001^c^	0.14^a^ (0.04, 0.24)	<0.001^c^
53–56.9	245	0.57^b^ (0.28, 0.86)		0.35^b^ (0.19, 0.51)		−0.01 (−0.13, 0.11)	
57–61.9	253	0.62^b^ (0.35, 0.89)		0.34^b^ (0.16, 0.52)		−0.06 (−0.16, 0.04)	
≥62	261	0.46^b^ (0.24, 0.68)		−0.25^a^ (−0.45, −0.05)		−0.25^b^ (−0.37, −0.13)	
BMI category, kg/m^2^							
<20	98	0.14 (−0.17, 0.45)	0.003^c^	0.69^b^ (0.44, 0.94)	<0.001^c^	0.24^b^ (0.12, 0.36)	<0.001^c^
20–24.9	692	0.45^b^ (0.29, 0.61)		0.37^b^ (0.27, 0.47)		0.03 (−0.03, 0.09)	
25–29.9	195	0.95^b^ (0.71, 1.19)		−0.23^a^ (−0.45, −0.01)		−0.41^b^ (−0.53, −0.29)	
≥30	25	0.40 (−0.42, 1.22)		−0.88^a^ (−1.59, −0.17)		−0.48 (−1.03, 0.07)	

Difference = (self-reported value − measured value).

^a^*P* < 0.05; ^b^*P* < 0.001; ^c^*P* for trend; ^d^*P* for difference calculated by ANOVA test; ^e^*P* for difference calculated by *t*-test.

Abbreviations: CI, confidence interval; BMI, body mass index.

Kappa values were reasonably high when the quartiles or 4 categorical groups of self-reported values were compared to measured values (Table 3). Kappa values for self-reported height and weight were 0.796 (95% CI: 0.756–0.836) and 0.839 (0.803–0.875), respectively, for men, and 0.746 (0.714–0.778) and 0.795 (0.765–0.825) for women. Kappa values were higher for BMI categories (<20, 20–24.9, 25–29.9, ≥30 kg/m^2^) than quartiles of BMI (0.78 vs. 0.732 in men; 0.789 vs. 0.727 in women). The prevalence of obesity based on self-reported values was 40.9% in men and 19.8% in women (Table 4). The prevalence of obesity based on self-reported values underestimated the true prevalence of obesity by 1.1% in men and 2.0% in women. Specificities were very high: 92.9% in men and 98.0% in women. Sensitivity was 87.6% in men and 83.6% in women.

**Table 3.** Number of subjects (percent) by cross-tabulation of quartile of self-reported and measured anthropometric indices

Self-reported	Measured				Kappa (95% CI)
Men (*n* = 557)					
Quartile of height, cm	<166	166–168.9	169–172.9	≥173	
<166	117 (87.3)	7 (6.7)	0 (0.0)	0 (0.0)	0.796^a^
166–168	15 (11.2)	68 (65.4)	9 (5.7)	0 (0.0)	(0.756, 0.836)
169–172	2 (1.5)	28 (26.9)	137 (86.2)	9 (5.6)	
≥173	0 (0.0)	1 (1.0)	13 (8.2)	151 (94.4)	

Quartile of weight, kg	<65	65–69.9	70–75.9	≥76	
<65	119 (95.2)	9 (6.7)	0 (0.0)	0 (0.0)	0.839^a^
65–69.9	6 (4.8)	110 (82.1)	7 (4.6)	0 (0.0)	(0.803, 0.875)
70–75.9	0 (0.0)	15 (11.2)	133 (86.9)	17 (11.7)	
≥76	0 (0.0)	0 (0.0)	13 (8.5)	128 (88.3)	

Quartile of BMI, kg/m^2^	<22.9	22.9–24.4	24.4–26.3	≥26.3	
<22.9	130 (91.6)	21 (15.2)	1 (0.7)	1 (0.7)	0.732^a^
22.9–24.4	12 (8.4)	96 (69.6)	18 (12.9)	1 (0.7)	(0.686, 0.776)
24.4–26.3	0 (0.0)	19 (13.8)	107 (77.0)	24 (17.4)	
≥26.3	0 (0.0)	2 (1.4)	13 (9.4)	112 (81.2)	

BMI category, kg/m^2^	<20	20–24.9	25–29.9	≥30	
<20	14 (66.7)	1 (0.3)	0 (0.0)	0 (0.0)	0.780^a^
20–24.9	7 (33.3)	278 (92.1)	28 (12.9)	1 (5.9)	(0.731, 0.829)
25–29.9	0 (0.0)	23 (7.6)	184 (84.8)	2 (11.8)	
≥30	0 (0.0)	0 (0.0)	5 (2.3)	14 (82.4)	

Women (*n* = 1010)					
Quartile of height, cm	<154	154–157.9	158–160.9	≥161	
<154	172 (80.4)	18 (6.3)	0 (0.0)	4 (1.5)	0.746^a^
154–157.9	39 (18.2)	197 (68.4)	10 (4.3)	0 (0.0)	(0.714, 0.778)
158–160.9	2 (0.9)	70 (24.3)	194 (83.3)	16 (5.8)	
≥161	1 (0.5)	3 (1.0)	29 (12.4)	255 (92.7)	

Quartile of weight, kg	<53	53–56.9	57–61.9	≥62	
<53	219 (87.3)	20 (8.2)	0 (0.0)	1 (0.4)	0.795^a^
53–56.9	31 (12.4)	179 (73.4)	14 (5.5)	0 (0.0)	(0.765, 0.825)
57–61.9	1 (0.4)	45 (18.4)	212 (83.8)	16 (6.1)	
≥62	0 (0.0)	0 (0.0)	27 (10.7)	244 (93.5)	

Quartile of BMI, kg/m^2^	<21.3	21.3–22.8	22.8–24.8	≥24.8	
<21.3	206 (81.1)	29 (11.3)	5 (2.0)	2 (0.8)	0.727^a^
21.3–22.8	45 (17.7)	191 (74.6)	32 (12.5)	0 (0.0)	(0.693, 0.760)
22.8–24.8	3 (1.2)	35 (13.7)	200 (78.4)	37 (15.1)	
≥24.8	0 (0.0)	1 (0.4)	18 (7.1)	206 (84.1)	

BMI category, kg/m^2^	<20	20–24.9	25–29.9	≥30	
<20	76 (77.6)	17 (2.5)	0 (0.0)	0 (0.0)	0.789^a^
20–24.9	22 (22.4)	659 (95.2)	36 (18.5)	0 (0.0)	(0.750, 0.828)
25–29.9	0 (0.0)	15 (2.2)	158 (81.0)	8 (32.0)	
≥30	0 (0.0)	1 (0.1)	1 (0.5)	17 (68.0)	

^a^*P* < 0.001.

Abbreviations: CI, confidence interval; BMI, body mass index.

**Table 4.** Prevalence of obesity based on self-reported and measured values and validity of self-reported anthropometric indices

	Men (*n* = 557)	Women (*n* = 1010)
Prevalence of obesity (BMI ≥25.0 kg/m^2^)		
Based on self-reported values (%)	40.9	19.8
Based on measured values (%)	42.0	21.8
Validity		
Sensitivity (%)	87.6	83.6
Specificity (%)	92.9	98.0
Positive predictive value (%)	89.9	92.0
Negative predictive value (%)	91.2	95.6

Abbreviations: CI, confidence interval; BMI, body mass index.

## DISCUSSION

Our analyses showed high rank correlations between self-reported and measured values for height, weight, and BMI in both men and women, demonstrating that self-reported data are reasonably valid substitutes for measured anthropometric indices in clinical and public health practice and biomedical research.

As compared with measured values, average self-reported height in men was 0.41 cm higher and weight was 0.12 kg higher; however, the difference in weight measurements was not statistically significant. A systematic review of 53 studies that assessed self-reported and measured height noted that height was overestimated in most studies.^25^ Men overestimated their height in 27 of 29 studies (range of overestimation, 0.1–5.0 cm); height was underestimated in only 2 studies (mean differences, −1.3 cm and −0.7 cm). Women overestimated their height in 26 of 30 studies (range, 0.3–5.0 cm); height was underestimated in only 4 studies (range, −1.7 to −0.1 cm). Therefore, our results are in line with previous studies, which showed overestimation of height among adult populations. Another Korean study, conducted by Song et al,^19^ reported that mean self-reported height was 1.36 cm higher than measured values.

Of 56 studies with data on both self-reported and measured weight, weight was underestimated in all studies (range, −2.7 to −0.1 kg) except 2, which noted mean differences of 0.3 kg and 0.6 kg. It is interesting that our study participants tended to overestimate their weight, in contrast to most other previous studies. However, the degree of overestimation decreased with increasing weight and BMI among both men and women. These findings are similar to those observed in studies conducted in British and Korean populations, which also reported overestimation of self-reported weight among women.^19^^,^^26^

Among 29 studies, BMI was underestimated in all (range, −1.8 to −0.2 kg/m^2^) but 2.^25^ Our study showed that BMI based on self-reported height and weight tended to be underestimated in both sexes, as compared with BMI based on measured values, although the difference between the 2 values was statistically significant only for men. Our findings were similar to those of a Korean study conducted by Song et al.^19^

The prevalence of obesity (ie, BMI ≥25.0 kg/m^2^) based on BMI calculated using self-reported values was lower than that based on BMI calculated using measured values. However, sensitivity and specificity were very high. Kappa values were also excellent (over 0.7), indicating substantial agreement (*P* < 0.001).^27^ A possible explanation for the excellent agreement between self-reported and measured values in our study population is that participants were expecting to be measured for weight and height during the physical examination, and this may have contributed to the accuracy of the self-reported values.

Many studies have investigated the association between age and bias in self-reported values, and the results have not been consistent.^12^^,^^14^^–^^19^^,^^28^ In most of these studies, differences in height, weight, and BMI increased with increasing age in men and women. In our analyses, age was associated with a difference between self-reported and measured height in men, and similar results have been observed in previous studies.^11^^,^^12^ Among women, age was associated with a difference between self-reported and measured weight. In previous studies, younger women tended to underestimate their weight more often.^12^^,^^13^^,^^16^^,^^18^ In addition, overestimation of self-reported height decreased with higher levels of educational attainment.^11^^,^^16^^,^^19^ A few studies have reported an association between chronic disease status and reporting biases in weight.^13^^,^^16^^,^^28^^,^^29^ Bolton-Smith et al reported that men with diabetes underestimated their weight more often than did those without diabetes, but there was no significant difference in women. These findings are similar to those of the present study.

Although our results should be interpreted with care because of the use of multiple comparisons, our findings provide useful insight into the use of self-reported anthropometric data. Caution should be exercised when using self-reported height and weight data, however, especially for groups with characteristics that predict biased reporting, such as individuals who are older or those with higher body weights. Although BMI calculated by using self-reported height and weight produced lower values than did calculations using measured values, self-reported height and weight data were shown to be reasonably valid in our study population and therefore could be used in clinical and public health settings when resources are limited.
